# Nitrogen physiology of contrasting genotypes of *Chenopodium quinoa* Willd. (Amaranthaceae)

**DOI:** 10.1038/s41598-018-34656-5

**Published:** 2018-11-30

**Authors:** Luisa Bascuñán-Godoy, Carolina Sanhueza, Katherine Pinto, Leonardo Cifuentes, María Reguera, Vilbett Briones, Andrés Zurita-Silva, Rodrigo Álvarez, Andrea Morales, Herman Silva

**Affiliations:** 1 Laboratorio de Fisiología Vegetal, Departamento de Botánica, Facultad de Ciencias Naturales y Oceanográficas, Universidad de Concepción, Casilla, 160-C Concepción Chile; 2 Instituto de Investigación Multidisciplinar en Ciencia y Tecnología, Universidad de La Serena, La Serena, Chile; 3 Laboratorio de Fisiología Vegetal, Centro de Estudios Avanzados en Zonas Áridas (CEAZA), La Serena, Chile; 4 Departamento de Biología, Universidad Autónoma de Madrid, Madrid, Spain; 5 Departamento de Ingeniería en Alimentos, Universidad de La Serena, La Serena, Chile; 6 Instituto de Investigaciones Agropecuarias, Centro de Investigación Intihuasi, La Serena, Chile; 7 Escuela de Tecnología Médica, Facultad de Salud, Sede La Serena, Universidad Santo Tomas, La Serena, Chile; 8 Laboratorio de Genómica Funcional y Bioinformática, Departamento de Producción Agrícola, Facultad de Ciencias Agronómicas, Universidad de Chile, Santiago, Chile

## Abstract

Quinoa has been highlighted as a promising crop to sustain food security. The selection of physiological traits that allow identification genotypes with high Nitrogen use efficiency (NUE) is a key factor to increase Quinoa cultivation. In order to unveil the underpinning mechanisms for N-stress tolerance in Quinoa, three genotypes with similar phenology, but different NUE were developed under high (HN) or low (LN) nitrogen conditions. N metabolism processes and photosynthetic performance were studied after anthesis and in correlation with productivity to identify principal traits related to NUE. We found that protein content, net photosynthesis and leaf dry-mass were determinant attributes for yield at both HN and LN conditions. Contrastingly, the enhancement of N related metabolites (${{\rm{NH}}}_{4}^{+}$, proline, betacyanins) and processes related with re-assimilation of ${{\rm{NH}}}_{4}^{+}$, including an increment of glutamine synthetase activity and up-regulation of *CqAMT1*,*1* transporter expression in leaves, were negatively correlated with grain yield at both N conditions. Biochemical aspects of photosynthesis and root biomass were traits exclusively associated with grain yield at LN. The impact of N supply on seed quality is discussed. These results provide new insights towards the understanding the N metabolism of Quinoa.

## Introduction

Nitrogen (N) is an essential mineral nutrient required by plants, and it is a constituent of distinct cellular components, including nucleic acids, proteins and amino acids. N is a determinant factor in all plant developmental stages, from seed germination to senescence and is considered a key factor limiting crop yield and quality^1^.

In the past half century, many crops varieties were selected to obtain maximum grain yield potential under high nitrogen input. However, the excessive use of nitrogen fertilizer resulted in a decreased nitrogen-use efficiency (NUE)^2^. In fact, only an average of 30–50% of the applied N is taken up by plants leading to extensive environmental pollution by N leaching^2^. Today N cost represents the highest budget item for farmers, therefore the improvement of N management and the use of cultivars/genotypes with high NUE is highly required.

N use efficiency has been defined in multiple ways; however, from an agronomical point of view, it can be defined as the yield produced per unit of N applied. NUE comprises both, firstly, the ability of the plant to take up N from the soil termed “nutrient uptake efficiency” and secondly the ability of the plant to transfer N to plant organs and yield, known as “nutrient utilization efficiency”^3^. Plants have evolved versatile mechanisms for N use increase. Changes in root biomass and architecture^3^, expression of high-affinity transporters (ammonium and nitrate), and enzymes related with primary assimilation such as nitrate reductase (NR), nitrite reductase (NiR), glutamine synthetase (GS) and glutamate dehydrogenase (GDH) which play a central role in efficient N assimilation under low N availability^4,5^.

During senescence, the disassembly of the photosynthetic apparatus determines nutrient recycling, re-assimilation and remobilization processes. Here, nutrients stored in RuBisCO and photosystem II (PSII) proteins from mature leaves are translocated to the remaining organs and seeds/grain of the plant. Low nitrogen supply and other stress conditions could induce accelerated senescence, reducing the time period for nutrients translocation and resulting in penalties on yield and quality^6^. During these conditions, high amounts of ammonium (${{\rm{NH}}}_{4}^{+}$) are released by different pathways (such as the enhancement of photorespiration, protein degradation and phenylpropanoid pathway) at rates that can exceed the rates of primary nitrate assimilation in plants^7^. These high ${{\rm{NH}}}_{4}^{+}$ levels are cytotoxic and consequently different physiological pathways may be induced in order to minimize injury and N loss^8^. It has been proposed that ${{\rm{NH}}}_{4}^{+}$ re-assimilation is a crucial pathway that contributes significantly to total N balance under limiting N conditions^6,9^. Glutamine synthetase (GS) catalyses the critical incorporation of inorganic ${{\rm{NH}}}_{4}^{+}$ into the amino acid glutamine (Gln)^10^. GS overexpression promoted physiological improvement on photosynthesis and growth at limiting N fertilization^6,9^. On the other hand, ${{\rm{NH}}}_{4}^{+}$ transporters in *Arabidopsis thaliana* participate in concentrative ${{\rm{NH}}}_{4}^{+}$ acquisition in roots, in long-distance transport to the shoots, and in re-uptake of apoplastic ${{\rm{NH}}}_{4}^{+}$ that derives from photorespiration in shoots. *AMT1;1*, a high-affinity ${{\rm{NH}}}_{4}^{+}$ transporter, is strongly de-repressed in response to plant N status variations, contributing to enhanced N balance through ${{\rm{NH}}}_{4}^{+}$ re-uptake in mesophyll cells^11^. An improved understanding of the mechanisms underpinning ${{\rm{NH}}}_{4}^{+}$ physiology would be vital for future NUE increases in crops.

Quinoa is considered a crop with the potential of contributing to food security worldwide^12^. Quinoa has exceptional nutritional properties of seeds, including elevated protein content and the good balance of essential amino acids^13^. In addition, it is able to withstand extreme environmental conditions such salinity and drought stress^14–16^. For all the above reasons, Quinoa production has undergone an exponential increment in the last decade, and its cultivation has been extended into many different areas of the world^15^. In general, Quinoa yield increases strongly in response to N fertilization supply^17–19^. However, a high N input is often not affordable for smallholder producers around the world. Within this context, it is desirable to identify varieties/genotypes with high tolerance to N limiting conditions.

Globally, there are more than 6000 landraces of Quinoa cultivated by farmers^15^. Those cultivars can be classified into five ecotypes according to their adaptation to specific agro-ecological conditions: Highlands (also known as Altiplano type); Inter-Andean Valleys; Yungas (grown under tropical conditions); Salares (grown at high altitude salt lakes areas and limited volume of annual rainfall (150–300 mm)) and Coastal/lowlands (where annual rainfall ranges from 500 to 1500 mm)^14,15^. Among these ecotypes, coastal/lowlands genotypes are of particular importance due to their photoperiod adaptation response that makes them highly suitable for spreading Quinoa cultivation into different climatic areas^20–22^. In fact, coastal Chilean genotypes have been used as elite parental sources in European Quinoa breeding programs^20,21^ and a coastal Chilean genotype was used for the Quinoa genome sequencing project^23^. Nevertheless, genotypes from different coastal/low land regions of Chile exhibited high phenotypic variability, differential agronomical performance and tolerance to stress conditions^24–26^. Also, these studies have demonstrated genotype dependent responses to specific stresses. Genotypic differences in NUE have been reported for a number of crops species, however, much less is known about NUE of different Quinoa genotypes^17,18,27,28^. We think that the wide Chilean Quinoa variability represents an important resource for selection NUE genotypes suitable for growing under different edaphoclimatic, soil and nutrients conditions.

In this work we prompt to define the physiological responses of Quinoa genotypes with different NUE, in order to address the best physiological and agronomical indicators of yielding at LN supply. The new information provided here will supply breeders about N dynamics in Quinoa for future improving programs.

## Results

### Impact of N regime on yield and NUE among Quinoa genotypes

**Figure 1:**
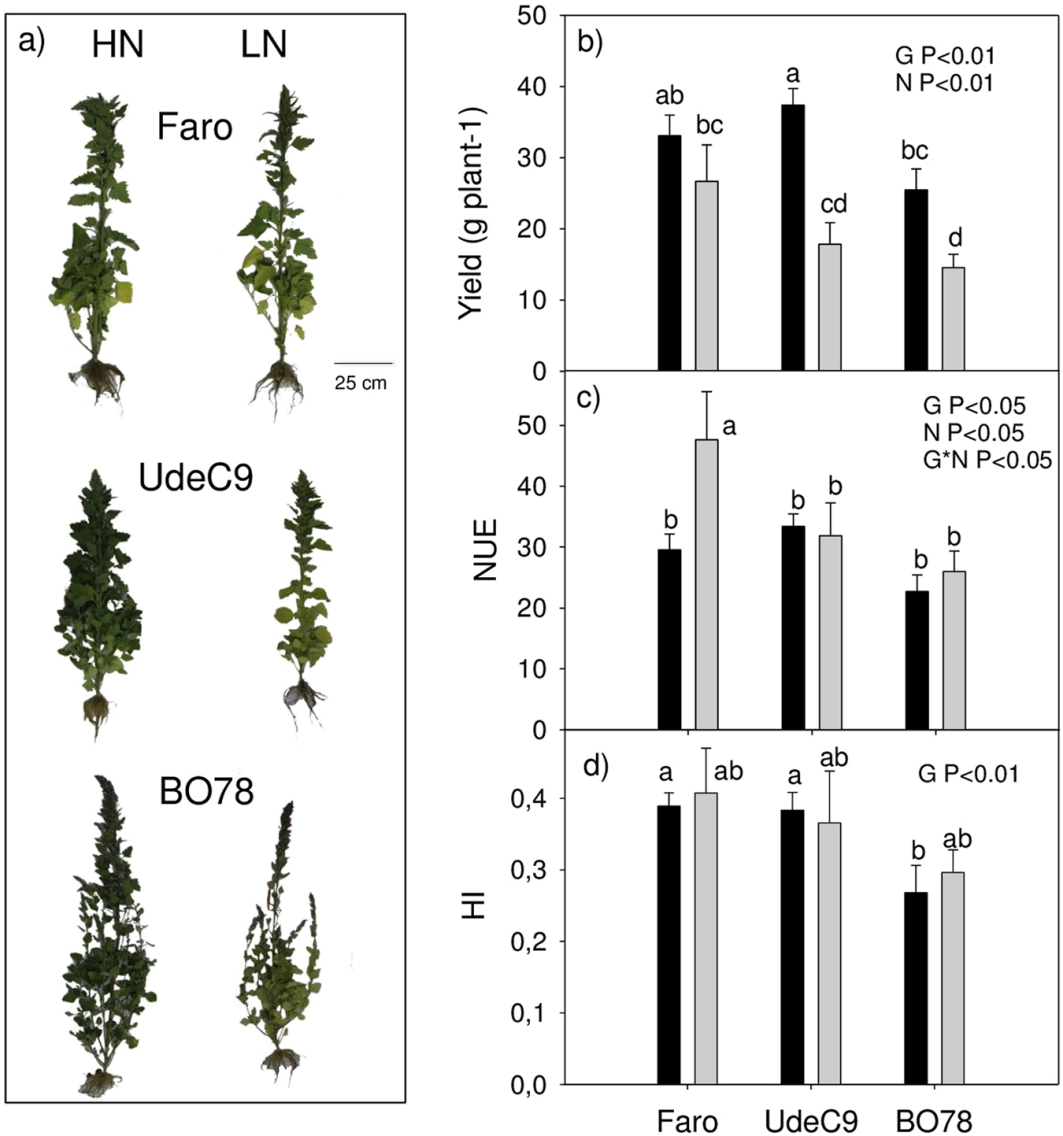
Phenotype, Yield, Nitrogen Use Efficiency (NUE) and Harvest Index (HI) of three genotypes of *C*. *quinoa* growing under different Nitrogen supplies. (**a**) Four-month-old Faro (top), UdeC9 (middle) and BO78 (botton) were grown at High Nitrogen (HN) and Low Nitrogen (LN) supplies. Photographs were taken two weeks after flowering. (**b**) Yield (**c**) NUE (**d**) HI. Bars show Mean values ± SE (*n* = *4*). Different letters represent significant differences among genotypes and treatments at *P* < *0*.*05* using two-way ANOVA.

In general, a most robust phenotype was observed in plants grown at HN than LN (Fig. 1a). Yield was affected by G (*P* < *0*.*01*) and N (*P* < *0*.*01*) (Fig. 1b). Under HN conditions, UdeC9 was the most productive genotype followed by Faro and BO78. However, LN conditions reduced yield significantly in UdeC9 (>50% reduction) and in BO78 (40% reduction), while Faro remained unchanged. Therefore, a significant increase of 50% in NUE was observed for the Faro genotype under this last condition (Fig. 1c). Contrasting, the Harvest index (HI) was maintained among genotypes independent of N treatment (Fig. 1d).

### Biomass under different N supplies

Under HN conditions BO78 displayed smaller and thinner leaves than Faro and UdeC9 (*P* < *0*.*05*) (Table 1). Other biometric parameters such as biomass of total leaves, shoot and root were similar among genotypes at HN (*P* > 0.05). LN supply affected significantly the majority of structural traits evaluated in BO78 and UdeC9 genotypes (*P* < 0.05). LN strongly reduced leaf area, total leaves biomass weight and shoot weight, in both UdeC9 and BO78. Additionally, changes in shoot/root ratio were observed in BO78. At LN all genotypes displayed a lower root biomass compared to HN (N, *P* < 0.001). Roots were reduced significantly in 52%, 66% and 89% in Faro, UdeC9 and BO78, respectively.

**Table 1:** Biomass under different N supplementation conditions in three genotypes of *C*. *quinoa*.

Genotype	LAi (cm^2^)	SLA (cm^2^/g)	Total leaves weight (g)	Root (g)	Shoot (g)	Shoot/root
*Faro*						
HN	67 ± 8 (a)	145 ± 9 (c)	32 ± 4 (a)	17 ± 6 (a)	86 ± 7 (ab)	7 ± 2 (bc)
LN	66 ± 6 (a)	166 ± 13 (c)	27 ± 4 (a)	8 ± 2 (b)	69 ± 12 (b)	10 ± 2 (bc)
*UdeC9*						
HN	63 ± 2 (a)	174 ± 9 (c)	31 ± 4 (a)	11 ± 3 (ab)	78 ± 7 (ab)	8 ± 2 (bc)
LN	41 ± 5 (b)	171 ± 9 (c)	16 ± 2 (bc)	3.7 ± 0.5 (c)	45 ± 4 (c)	12 ± 1 (b)
*BO78*						
HN	35 ± 3 (b)	277 ± 13 (a)	23 ± 2 (ab)	18 ± 4 (a)	92 ± 4 (a)	6 ± 1 (c)
LN	19 ± 2 (c)	224 ± 15 (b)	8 ± 1 (c)	1.9 ± 0.3 (c)	39 ± 1 (c)	21 ± 3 (a)
G	<0.05	<0.05	n.s.	n.s.	<0.05	n.s
N	<0.001	n.s	<0.001	<0.001	<0.001	<0.05
G*N	n.s	n.s	n.s	n.s	n.s	n.s

Different parameters were determined to evaluate biomass changes associated with different N supplied. Fully expanded third leaves (from the top part of the plant) were used for individual leaf area measurements (LAi, cm^2^) and ratio of leaf area to dry mass (SLA, cm^2^/g) was also determined. Biomass of four different individuals (n = 4) are expressed as dry weight (DW). Different letters represent significant differences between genotypes (G; Faro, UdeC9 and BO78) and nitrogen supplementation (N; HN (high nitrogen) and LN (low nitrogen)) (*P* < 0.05) using two-way ANOVA. Shoot: root ratio was calculated for every single plant. The three last rows of the table show the significance levels (*P*) and interactions of the factors (G, N and G*N) for the parameters. n.s. = no significant.

### Changes in Chlorophyll content and chlorophyll a fluorescence under HN and LN conditions

BO78 showed a 50% lower level of both Chlorophylls (*a* and *b*) compared to Faro and UdeC9 at HN (Table 2). Significant reductions under LN were observed in UdeC9 and BO78 but not in Faro. UdeC9 showed the greatest decrease in both Chl *a* and *b* showing the highest Chl *a/b* ratio among studied genotypes (Table 2).

**Table 2:** Chlorophyll quantification in three genotypes of *C*. *quinoa* at different N supplies.

Pigments	Faro		UdeC9		BO78		G	N	G*N
	HN	LN	HN	LN	HN	LN			
Chl *a*	3.7 ± 0.9 (a)	2.2 ± 0.5 (abc)	3.3 ± 0.9 (ab)	0.5 ± 0.1 (d)	1.7 ± 0.3 (bc)	1.0 ± 0.3 (c)	n.s	0.005	n.s
Chl *b*	0.98 ± 0.25 (a)	0.54 ± 0.14 (abc)	0.75 ± 0.21 (ab)	0.08 ± 0.03 (d)	0.41 ± 0.09 (bc)	0.21 ± 0.08 (c)	0.05	0.005	n.s
Chl *a* + *b*	4.7 ± 1.2 (a)	2.7 ± 0.6 (abc)	4.0 ± 1.2 (ab)	0.5 ± 0.2 (d)	2.1 ± 0.4 (bc)	1.2 ± 0.4 (c)	0.05	0.005	n.s
Chl *a*/*b*	3.8 ± 0.1 (e)	4.0 ± 0.1 (ed)	4.4 ± 0.1 (c)	5.9 ± 0.1 (a)	4.2 ± 0.1 (cd)	4.9 ± 0.1 (b)	0.05	0.005	n.s

Leaf samples of three individual plants (*n = *3) were collected from each genotype at midday. Absolute quantities of chlorophylls (Chl) are expressed in µmol g^−1^ per FW. Analysis using a two way ANOVA followed by Tukey test was used to compare genotypes (G) and nitrogen treatments (N). Different letters represent significant differences between G (Faro, UdeC9 and BO78) and N (HN (high nitrogen) and LN (low nitrogen)). The three last rows of the table show the significance levels (*P*) and interactions of the factors (G, N and G*N) for the parameters. n.s. = no significant.

### N supplementation impact on photosynthesis

**Figure 2:**
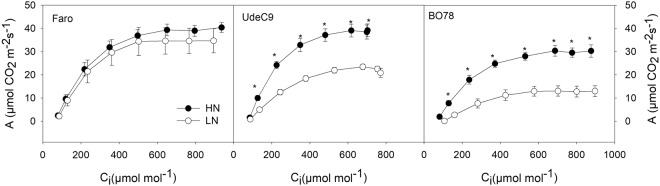
A/Ci curves [net CO_2_ assimilation rate (**A**) versus CO_2_ concentration (Ci)] of three genotypes of *Chenopodium quinoa* growing under different N supplies. Fully expanded third leaves (from the top) were used for the photosynthetic measurements two weeks after flowering. A/Ci curves of (**a**) Faro, (**b**) UdeC9 and (**c**) BO78 are shown. Values are mean ± SE (*n* = *4*). Significant differences between N supply within a genotype are indicated by asterisks at a *P* < *0*.*05* using one-way ANOVA.

At HN conditions, BO78 displayed a 25% lower level of net CO_2_ assimilation rate (A) (or net photosynthetic rate (*Pn*)) compared to Faro and UdeC9 genotypes (Fig. 2; Table 3). However, other photosynthetic parameters including *gs*, WUEi, A_max_, V_Cmax_, J_max_, TPU and CCP were similar among all studied genotypes. With the exception of WUEi, that remained constant despite N or genotype, LN growing conditions highlighted the differential capacity of each studied genotype to maintain photosynthetic parameters under this stressful condition. While both UdeC9 and BO78 genotypes showed a significant reduction in all photosynthetic parameters analyzed, Faro displayed photosynthetic parameter values similar to those obtained under HN conditions (Fig. 2; Table 3).

**Table 3:** Photosynthetic parameters determined in three lowland genotypes of *C*. *quinoa* subjected to HN and LN supplies.

	Faro		UdeC9		BO78		G	N	G*N
	HN	LN	HN	LN	HN	LN			
*Pn*	22 ± 3 (a)	20 ± 4 (ab)	21 ± 2 (a)	12 ± 1 (bc)	16 ± 2 (b)	8 ± 2 (c)	<0.05	<0.01	ns
*gs*	0.2 ± 0.04 (ab)	0.2 ± 0.05 (ab)	0.3 ± 0.05 (a)	0.16 ± 0.03 (b)	0.2 ± 0.04 (ab)	0.14 ± 0.04 (b)	<0.05	ns	ns
WUE i	200 ± 44 (a)	172 ± 32 (a)	143 ± 25 (a)	183 ± 38 (a)	200 ± 76 (a)	129 ± 29 (a)	ns	ns	ns
A _max_	41 ± 2 (a)	36 ± 5 (a)	40 ± 1 (a)	25 ± 1 (b)	34 ± 5 (ab)	14 ± 2 (c)	<0.01	<0.001	ns
V_Cmax_	55 ± 3 (a)	53 ± 6 (a)	55 ± 2 (a)	42 ± 1 (bc)	49 ± 2 (ab)	35 ± 2 (c)	<0.005	<0.005	ns
J_max_	286 ± 20 (a)	267 ± 44 (a)	271 ± 23 (a)	184 ± 7 (b)	224 ± 11 (a)	139 ± 11 (c)	<0.005	<0.005	ns
J_max_:V_Cmax_	5.2 ± 0.1 (a)	5.0 ± 0.3 (ab)	4.9 ± 0.3 (ab)	4.4 ± 0.1 (ab)	4.6 ± 0.2 (ab)	4.2 ± 0.1 (b)	<0.01	ns	ns
TPU	20 ± 1 (a)	19 ± 2 (a)	19 ± 1 (a)	15 ± 1 (b)	17 ± 1 (ab)	11 ± 1 (c)	<0.005	<0.005	ns
CCP	69 ± 8 (b)	85 ± 9 (ab)	79 ± 5 (b)	82 ± 3 (b)	71 ± 4 (b)	110 ± 18 (a)	ns	<0.05	ns

Net photosynthetic rate (*Pn*, μmol m^−2^ s^−1^), stomatal conductance (*g*_*s*_. mol m^−2^ s^−1^), intrinsic water-use efficiency (WUEi) were obtained at 400 μmol CO_2_ m^−2^ s^−1^. Maximum photosynthesis rate (A_max_), maximum rate of carboxylation (V_Cmax_) (μmol CO_2_ m^−2^ s^−1^), maximum rate of electron transport (J_max_) (μmol e^−^ m^−2^ s^−1^), use of trioses (TPU) (μmol Pi m^−2^ s^−1^) and CO_2_ Compensation point (CCP µmol mol^−1^) were estimated from the A/Ci curves obtained from the third fully expanded leaf using Photosyn Assistant software. Values are mean ± SE (n = 4). Different letters represent significant differences between genotypes (G; Faro, UdeC9 and BO78) and nitrogen supplementation (N; HN (high nitrogen) and LN (low nitrogen)) (*P* < 0.05) using two-way ANOVA. The three last rows of the table show the significance levels (*P*) and interactions of the factors (G, N and G*N) for the parameters. n.s. = no significant.

### N supply effects on N metabolism

**Figure 3:**
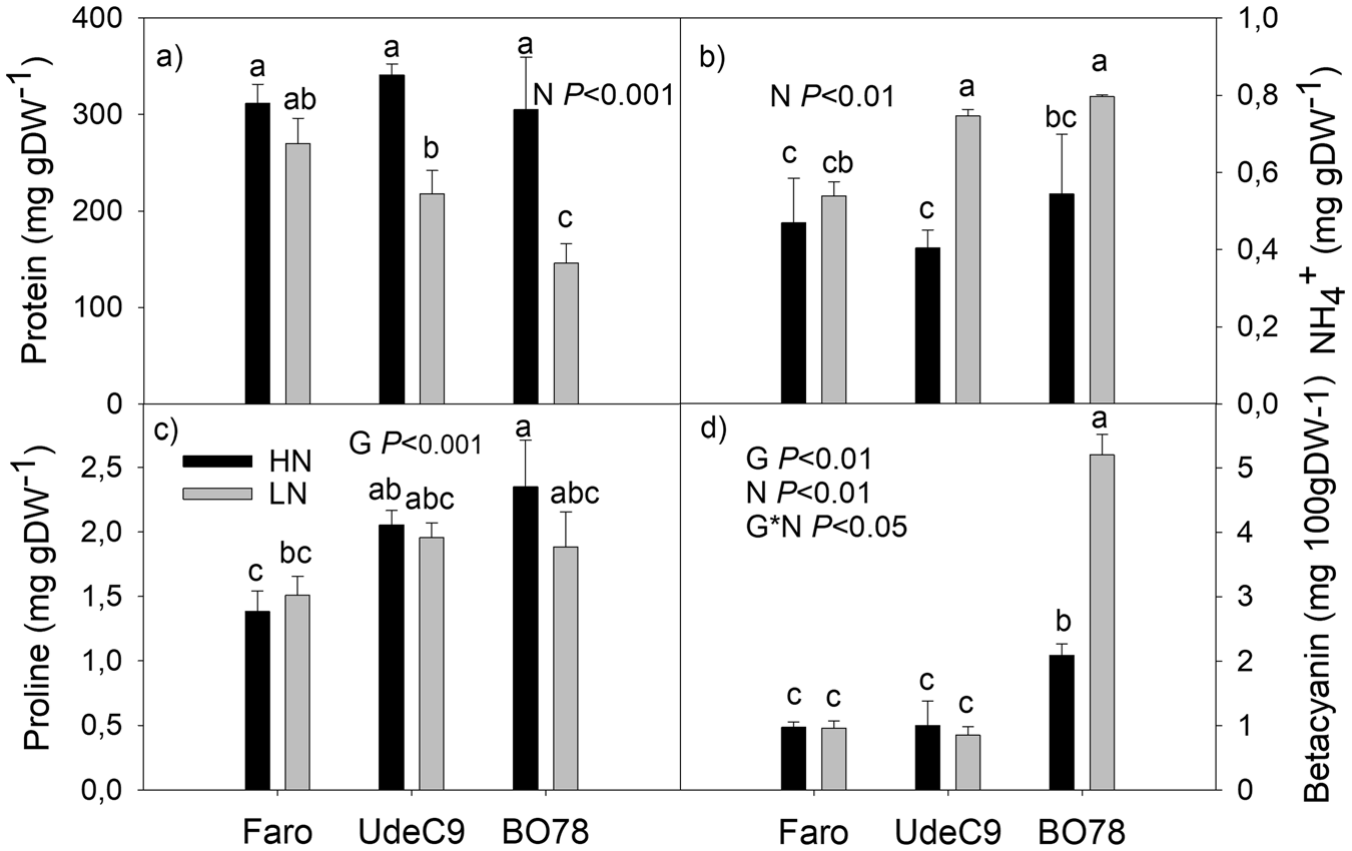
Changes in protein, ammonium (${{\rm{NH}}}_{4}^{+}$), proline and betacyanin contents in response to LN supply in three Quinoa genotypes. Fully expanded third leaves (from the top) were measured. Different letters indicate significant differences among genotypes and treatments at a *P* < 0.05 using two-way ANOVA. Values are mean ± SE (*n* = 4).

Protein content and ${{\rm{NH}}}_{4}^{+}$ concentrations were similar among all studied genotypes under HN conditions (Fig. 3a). During LN, however, both UdeC9 and BO78 genotypes displayed a reduction in total protein content and an increase in their NH4+ levels. The Faro genotype, on the contrary, maintained similar values of protein and NH4+ levels to those obtained at HN conditions (Fig. 3) highlighting once again its capacity to adapt to N stress.

**Figure 4:**
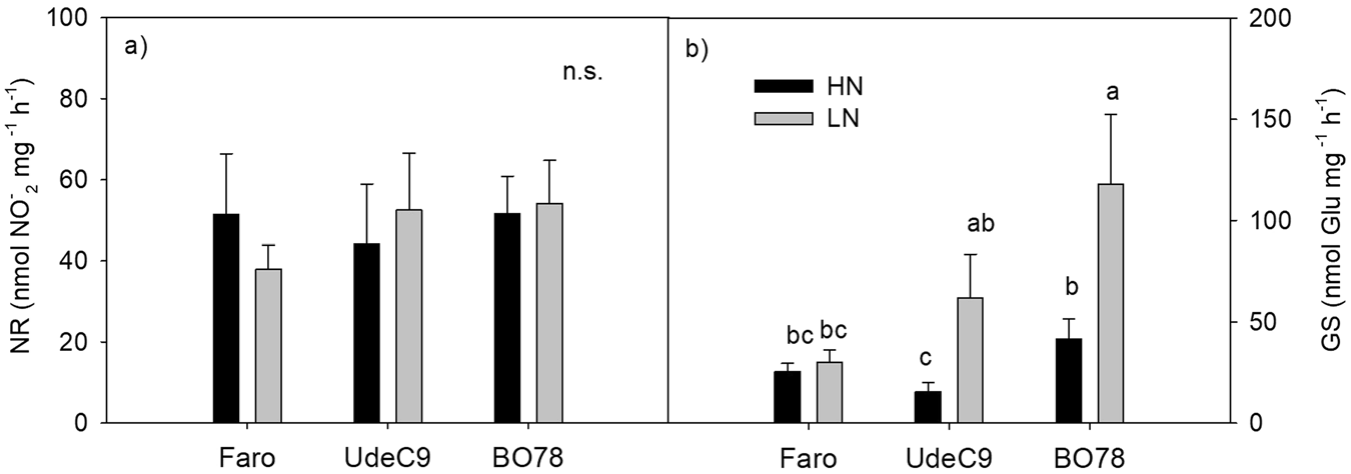
Changes in Nitrate reductase (NR) and Glutamine synthetase (GS) enzymatic activities in leaves of *C*. *quinoa* growing under different N supplies. Enzyme activities are expressed as mol of metabolite generated (${{\rm{NO}}}_{2}^{-}$ and γ-glutamyl hydroxamate for NR and GS respectively) per mg of protein per unit of time. Additional details are provided in the Methods section. Values are mean ± SE (*n* = 4). Different letters indicate significant differences among genotypes and treatments using a two-way ANOVA at a *P* < 0.05.

Differences in proline and betacyanin concentrations were detected among the studied genotypes under HN conditions, being BO78 the genotype showing the highest levels of both metabolites (Fig. 3c,d). Further, BO78 genotype showed a significant increase in betacyanin accumulation under LN conditions. Betacyanin concentrations depended on G, N and their interaction. Regard enzymes, NR activity remained unchanged among genotypes and N treatments (Fig. 4a); however, GS activity was significantly increased in UdeC9 and BO78 when grown at LN (Fig. 4b) (N *P* < 0.05; Fig. 3b).

### Expression changes of N metabolism-related genes in response to limited N

**Figure 5:**
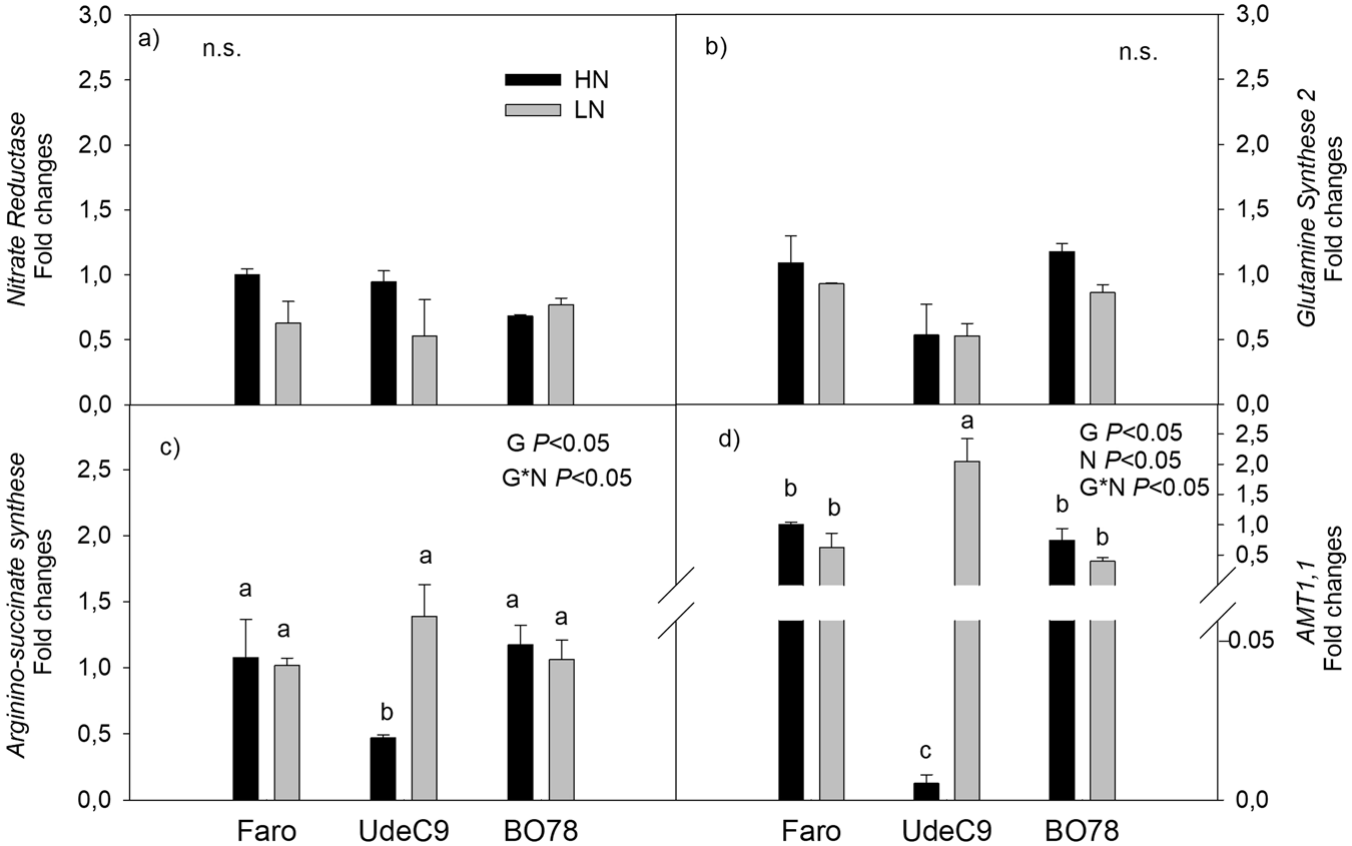
Expression levels of NH4+ reassimilation-related genes in leaves of three *C*. *quinoa* genotypes growing under different N supplies. Expression levels of (**a**) Nitrate Reductase (*CqNR*), (**b**) Glutamine synthetase 2 (*CqGS2*), (**c**) Argininosuccinate synthase 1 (*CqASS1*), (**d**) *AMT1* ammonium transporter (*CqAMT1*.*1*) were detected by quantitative PCR. Relative expression in Faro HN was used as reference. *CqHK1* was used as housekeeping. Bars show Mean values ± SE (n = 3). Letters indicate significant differences at a *P* < *0*.*05* in gene expression levels among genotypes and treatments (*P* < *0*.*05*) using two-way ANOVA.

Regarding the genes related to ${{\rm{NH}}}_{4}^{+}$ metabolism, no changes in CqNR or CqGS2 expression were detected among genotypes under either condition (Fig. 5). Furthermore, differential N supply induced similar expression patterns for *CqASS1* and *CqAMT1*,*1* in all genotypes studied. Under HN conditions, the UdeC9 genotype exhibited the lowest expression levels for *CqASS1* and *CqAMT1*,*1* when compared to Faro and BO78 genotypes. LN conditions induced an increase in the expression for these genes in all genotypes, however, the UdeC9 genotype presented the highest levels of expression showing a 3 fold increase for *CqASS1* and a 50 fold increase for *CqAMT1*,*1* (Fig. 5d) when compared to HN expression levels.

### LN effect on seed-related parameters and free amino acids pool in Quinoa

**Figure 6:**
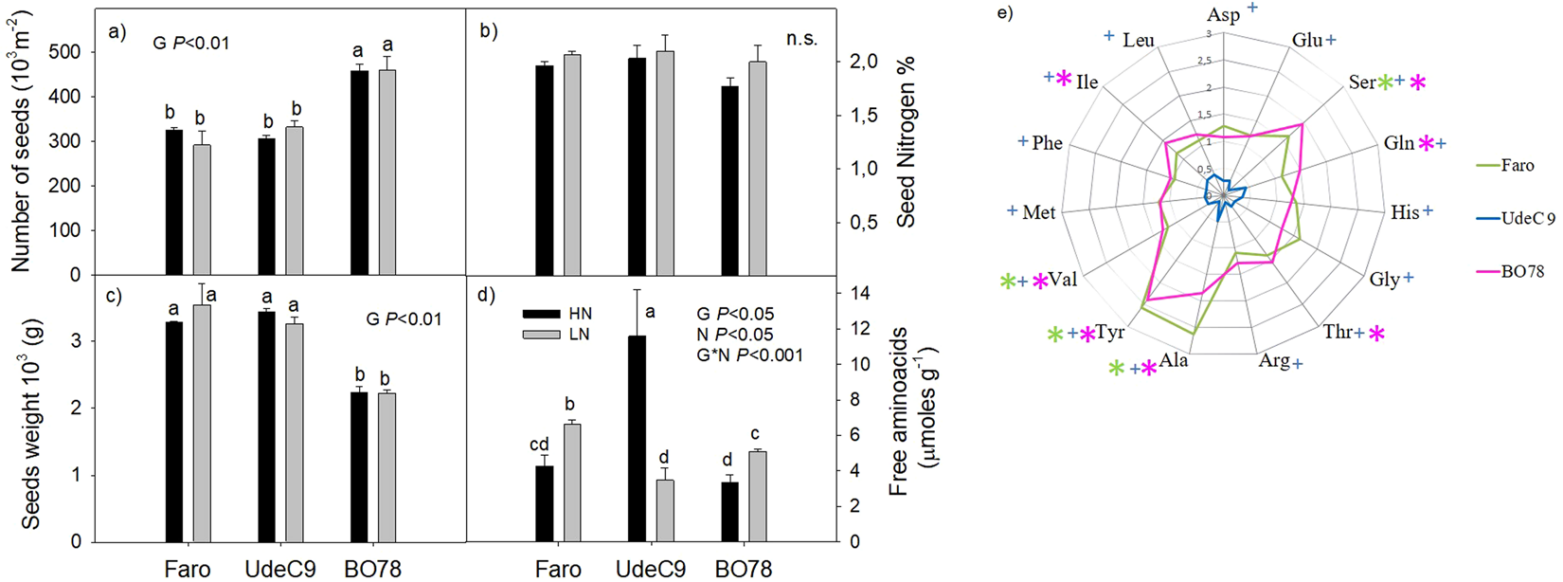
Seed-related parameters, nitrogen content and free amino acids pool in Quinoa subjected to different N supplies. (**a**) Number of seeds per m^2^, (**b**) seed N content (%), (**c**) weight of 1000 seeds and (**d**) total amino acid contents were determined in each genotype growing at two different N conditions. Bars show mean values ± SE (*n* = *4*). Different letters represent significant differences among genotypes and treatments at *P* < *0*.*05* using two-way ANOVA. (**d**) Radar chart shows relative changes in free amino acids in three genotypes of Quinoa that were calculated as the ratio of LN content to HN content. Changes observed between genotypes were denoted by different colors: Faro (green), UdeC9 (blue) and BO78 (pink). Asterisks/crosses (symbols) indicate significant increase/decrease of the amino acid respectively, when comparing LN/HN treatments per genotype. The lack of symbol indicates non significant differences between N conditions by each genotype.

Statistical differences in seeds were indeed observed under HN conditions among genotypes (*P* < *0*.*05*). BO78 presented the highest seed number per area and lowest seed weight among genotypes. Seed nitrogen content was similar among genotypes, although the free amino acid composition of seeds showed to be genotype dependent with UdeC9 exhibiting the highest levels of free amino acids and the largest differences in concentration were observed between genotypes UdeC9 and BO78. We did not observe any effect of LN conditions on seed number per area, seed weight or seed nitrogen content (Fig. 6a–c). LN conditions showed to have a detrimental effect on free amino acid content in UdeC9, in contrast, LN conditions induced an increase in free amino acid content in both Faro and BO78 genotypes reaching free amino acid content levels even higher than that present in UdeC9 genotype.

Relative changes of free amino acid contents were evaluated as the ratio of the amino acid content in LN seeds compared to HN seeds (Fig. 6e). Among the changes observed, all amino acids were significantly decreased under LN in UdeC9, which correlated well with the sharp decrease observed in the total amino acid pool (Fig. 6d). A different response was shown by Faro and BO78 that increased significantly contents of serine (Ser), alanine (Ala), tyrosine (Tyr) and valine (Val) under LN. Moreover, BO78 increased contents of threonine (Thr), glutamine (Gln) and isoleucine (Ile) under LN conditions (Fig. 6e).

### Correlations between grain yield and agronomical and physiological traits at different N supply

Pearson correlations values (*r*) between yield and physiological traits varied according to the N supply were determined (Table 4). Grain yield was positive and significant correlated with LAi, leaves biomass and *Pn* at both N regimens. Moreover, at HN but not at LN grain yield was highly positive significant correlated with seed weight, %N, proteins and amino acid in seeds and negative significant correlated with shoot biomass, betacyanin content and number of seeds m^−2^. Whereas, at LN but not at HN grain yield was highly positive significant correlated with roots biomass, *gs*, Chls, and A_max_, V_Cmax_, J_max_, J_max:_ V_Cmax_, TPU and negative significant correlated with proline, ${{\rm{NH}}}_{4}^{+}$ and GS.

**Table 4:** Correlation analysis of yield and different physiological traits under two different N supplies.

	LAi	SLA	Roots	Leaves	Shoot: root	Chl *a*	Chl *b*	Chl *a*/*b*
HN	**0**.**8**	−0.3	−0.22	**0**.**73**	0.38	0.49	0.43	0.36
LN	**0**.**72**	−0.6	**0**.**71**	**0**.**74**	0.014	**0**.**68**	**0**.**7**	−0.63
	**Pn**	**gs**	**WUEi**	**Amax**	**Jmax**	**Jmax: VCmax**	**TPU**	**CCpoint**
HN	**0**.**84**	0.33	0.64	0.38	0.65	0.44	0.52	0.16
LN	**0**.**94**	**0**.**89**	0.15	**0**.**74**	**0**.**84**	**0**.**72**	**0**.**91**	−0.33
	**Protein**	**NH4+**	**Proline**	**Betacyanin**	***CqNR***	***CqGS2***	***CqASS1***	***CqAMT1***.***1***
HN	**0**.**73**	−0.64	−0.51	**−0**.**73**	−0.56	−0.63	−0.38	−0.32
LN	**0**.**9**	**−0**.**92**	**−0**.**76**	−0.56	−0.45	**−0**.**74**	−0.41	**−0**.**62**
	**Seed weight**	**N° of seeds m** ^**−2**^	**% N seeds**	**Total free amino acids**				
HN	**0**.**69**	**−0**.**72**	**0**.**69**	**0**.**67**				
LN	0.31	−0.4	0.21	0.45				

The parameters analyzed under two different N supplies (HN (high nitrogen) and LN (low nitrogen)) included: individual leaf area [LAi], specific leaf area [SLA]), biomass dry weight (leaves, root, and shoot:root ratio), pigments (including chlorophyll *a* [Chl *a*], chlorophyll *b* [Chl *b*] and Chl *a/b*), photosynthetic parameters (including *Pn* [net photosynthesis], stomatal conductance [*gs*], intrinsic water use efficiency [WUEi]), maximum photosynthesis rate [A], maximum rate of carboxylation [V_Cmax_], maximum rate of electron transport [Jmax], Jmax:V_Cmax_ ratio, use of trioses [TPU]) protein, ${{\rm{NH}}}_{4}^{+}$ and proline contents, enzyme activities (NR and GS), relative gene expression of *CqAMT1*.*1* and yield and seed-related parameters (including harvest index [HI], seed weight, number of seed per area, N and protein contents and total free amino acids). Pearson correlation coefficient (PCC) was calculated; bold numbers denote significant correlation at a *P* < *0*.*05* and underlined numbers significant correlation at *P* < *0*.*001* (n = 12).

## Discussion

Considering the importance of Chilean coastal/lowland germoplasm for the cultivation of Quinoa, the comprehension of the physiological and molecular mechanisms that trigger adaptive responses to N deficit, particularly those involved in maintaining yield at LN availability are of crucial importance.

Our results confirmed a differential ability to respond to N deficit among the studied genotypes (Fig. 1a–d). At LN conditions, the Faro genotype experimented only a slight reduction in yield contrasting with the responses of both the UdeC9 and the BO78 genotypes that experimented an important reduction accounting for approximately 50% their yield obtained under HN conditions (Fig. 1c). Consequently, Faro showed to be the only genotype of this study able to increase NUE under LN conditions.

There is known that N is a strong determinant of total plant biomass, as confirmed in our present results (Table 1). There are some traits, such as LAi and total dry leaf mass, which positively correlate to yield independently of the N conditions. Root biomass, however, showed to be determinant for yielding only at LN conditions. It has been reported that N fertilization influence in the biomass, morphology and branching of roots^29^. We suggest that at LN the larger root development of Faro compared to UdeC9 and BO78 (Table 1) might help to an enhanced the uptake of nutrients creating a positive feedback between N status and growth. This, in turn, could lead to increasing leaf area and thickness and consequently plant yield (Table 1).

Contrastingly to Faro, which was able to maintain Chl*a* and *b* at LN, UdeC9 displayed the most remarkable reduction of these pigments to similar levels of BO78 (Table 2). Both UdeC9 and BO78 increased significant the values of Chl *a/b* ratio, indicating an enhanced degradation of the antenna complex capturing light. This could be seen as a photoprotective strategy to reduce the excess of light absorbed under conditions of stress and down regulation of the photosynthesis^30^. In the same way the increase of betacyanins induced in BO78 might have a protective role of photosystem II via attenuation of potentially harmful excess incident light^31^.

UdeC9 and BO78 also shown the largest *Pn* and stomatal conductivity (*gs*) reduction under LN compared to HN supplied plants (Fig. 2; Table 3). This response was indicating that restriction of CO_2_ stomata entry could be an important factor contributing to the high decrease of *Pn* in these genotypes (Table 3). In addition, when analyzing the A/Ci plot we found statistically significant differences between photosynthetic rates among genotypes at a given substomatal CO_2_ concentration. Also, we observed a significant reduction of biochemical CO_2_ fixation parameters: V_Cmax_, J_max_, and TPU in both UdeC9 and BO78, but not in Faro. These decreases are a common response to N deficiency after anthesis^32^ and these results, taken together, denote a differential photosynthetic performance among genotypes.

It has been reported that J_*max*_:V_Cmax_ relationship is maintained tight across growth environments and species^33^. In accordance, our results shown that LN affected similarly J_*max*_ and V_Cmax_ level (Table 3) indicating that N resource allocation on electron transport is reduced to couple to Calvin–Benson cycle decay under LN conditions. We suggest that this could be a strategy to decrease the cost for dissipation of that energy which would not be used on photosynthesis and then reduce the probability of ROS production on the electron transport chain^34^.

According with the reduction of photosynthetic performance (*Pn*, *g*_*s*_, V_Cmax_) and the enhancement of the CO_2_ compensation point (CCP) value (Table 3), we found an increment of ${{\rm{NH}}}_{4}^{+}$ in both UdeC9 and BO78 genotypes (Fig. 3). The CCP is used as an estimation of photorespiration, a process that releases great quantities of ${{\rm{NH}}}_{4}^{+}$^35^. Photorespiration is an alternative electron sink under stress conditions^36^ and has a protective role for survival under limiting N status sensing, as has been already proposed by Fuentes *et al*., and Masclaux-Daubresse *et al*.^6,9^. In the case of BO78, we cannot exclude that other processes such as protein degradation and/or the induction of the shikimate pathway could be also contributing to ${{\rm{NH}}}_{4}^{+}$ accumulation. However, the important increment observed in CCP suggests that this alternative process is important to avoid over-reduction of the electron transport chain in this genotype.

The increase of GS activity in UdeC9 and BO78 (but not NR) (Fig. 4) and the up-regulation of the expression level of *CqASS1* and *CqAMT1*,*1* in UdeC9 (Fig. 5) indicate that processes related with ${{\rm{NH}}}_{4}^{+}$ re-uptake are markedly more expressed at LN compared to HN conditions in these genotypes.

*ASS1*, that codifies the argininosuccinate synthase enzyme (ASS), catalyzes one of the rate-limiting steps in Arg biosynthesis. Arg is one of the main amino acids that act as an innocuous reservoir of ${{\rm{NH}}}_{4}^{+}$ in Quinoa leaves^25^. On the other hand, *AMT1*,*1* in *Arabidopsis thaliana* leaves participates in the reuptake of apoplastic ${{\rm{NH}}}_{4}^{+}$ (that could be lost as gas in leaf mesophyll cells), thus, contributing to a positive C/N balance^11^.

Surprisingly, we found a strong negative correlation between yield and ${{\rm{NH}}}_{4}^{+}$, GS activity, *ASS1* and *AMT1*,*1* gene expression at both HN and LN conditions. Our results are consistent with those reported for finger millet, where high NUE genotypes presented a low induction of the ${{\rm{NH}}}_{4}^{+}$ assimilation pathway^37^. These results suggest that ${{\rm{NH}}}_{4}^{+}$ reuptake constitutes a mechanism used mostly by sensitive plants to ameliorate the increased levels of ${{\rm{NH}}}_{4}^{+}$ derived from different physiological processes after anthesis.

Finally, in order to observe if the induction of ${{\rm{NH}}}_{4}^{+}$ reuptake in leaves induced any changes in seeds we studied several seed characteristics including total N content and free amino acids (Fig. 6). It was noteworthy that all genotypes were able to maintain the size, weight and N content of their seeds, which was consistent with Alandia *et al*.^28^ working on a series of N treatments on Quinoa Titicaca cv.

Despite the results showing no effect on seed N content, sharp changes in the total free amino acid pool were observed in all genotypes studied under different N conditions (Fig. 6d). UdeC9 presented a strong decrease in the majority of amino acids but BO78 and Faro genotypes showed an increase in the general pool of free amino acids. The positive correlations between %N and total free amino acids in seeds and yield suggested that the translocation of resources was more limited for yielding at HN than at LN. This is in agreement with several studies that compared the capacity to remobilize resources in limiting *vs* sufficient supplied plants^6^.

Among the highly increased amino acids in Faro and BO78 was tyrosine (Tyr) and Alanine (Ala) (Fig. 6e). Tyr is a precursor of betacyanins^13,38,39^ and Ala, on the other hand, has been linked with a NUE phenotype in barley, canola and *Arabidopsis*^40,41^. The form how N is contained in seeds determines their nutritional quality, germination capability and seedling establishment, therefore it is of crucial importance to understand the impact N supply has on seed N composition. Our study addresses this issue, but further experiments are necessary to understand the role of N supply on the performance of next-generation plants.

Summarizing, limiting N conditions exalted the different abilities to maintain yield and quality among genotypes. The mechanisms associated with the ${{\rm{NH}}}_{4}^{+}$ reuptake were more related to the maintenance of cellular homeostasis in LN sensitive plants than the capacity to tolerate LN or to produce yield. The most relevant correlations with yield at both HN and LN were LAi, leaf biomass, P*n* and protein content. Instead, root biomass, Chl content, and the biochemical photosynthetic processes were traits determinant for yielding only at LN conditions.

Concluding, our results provide new physiological knowledge about the mechanisms underlying the differential NUE at LN in Quinoa and provide new traits to test for in breeding programs. The roots development emerges as a selective trait towards selecting varieties for poor soils.

## Material and Methods

### Plant material and growth conditions

Three lowland genotypes: Faro (latitude 34.47° and longitude 71.83°), UdeC9 (latitude 35.73° and longitude 72.53°) and BO78 (latitude 38,51° and longitude 71,4) from different geographical and climatic areas of Chile, but with similar morphological and phenological (senescing timing) characteristics, were used in this work. It has been reported that Faro increment its NUE value when developed at LN^27^, and display an enhanced level of photoprotective attributes when grown at LN compared with UdeC9 and BO78^42^. Faro seeds were obtained from Cooperative Las Nieves, whereas UdeC9 and BO78 seeds were provided for the National Seed Bank collection at Vicuña, Chile (INIA-Intihuasi).

Experiments were conducted in pots from September 2015 until February 2016 in a greenhouse. The environmental conditions were: 1,200 µmol m^−2^ s^−1^ PAR at noon (natural light), maximum and minimum temperatures (daily ranges) of 23 °C and 17 °C respectively, 12 h day length, and 80% relative humidity. Seeds were germinated directly in soil in pots of 10 L filled with equal amounts of dry soil (5 Kg). Soil composition consisted in a mixture of 80% sand and 20% peat. The nutrients were applied in one dose because N split previously showed to have only a weak effect on yield^43^. Nutrient contents were: N: 40 mg/kg; P: 96 mg/kg and K 690 mg/Kg. Soils were supplemented with urea (CH_4_N_2_O) to reach two N level treatments: high nitrogen soils (HN; 0.6 g of N per pot) and low nitrogen soils (LN; 0.30 g of N per pot). These concentrations were used considering the optimal and insufficient N fertilization levels reported for Quinoa^17,18^. Plants (one per pot of 10 L volume) were irrigated to field capacity every three days maintaining its optimal moisture soil, till seed maturation. The experiment was run as a completely randomized design and supplementary plants were used to prevent bordering effect. Measurements of biomass, proteins, ${{\rm{NH}}}_{4}^{+}$, enzymes activities, expression analysis, chlorophylls, and gas interchange were performed after two weeks of panicle initiation (December, grain filling stage). Measurements about yielding were performed at the end of the life cycle.

### Yield, NUE and HI

Grain yield was determined as the total grain weight per plant at the end of growth season. The Nitrogen Use efficiency (NUE) was calculated by dividing the seed yield by total N applied. The harvest index (HI) of each treatment was calculated from the ratio among seed yield and shoot dry matter.

### Leaf area and biomass

Individual leaf area (LAi) was measured with an area meter (CI-203, CID Bio-Science Inc, USA)^44^. Specific leaf area (SLA) was calculated as the ratio of leaf area to leaf dry mass (cm^2^ g^−1^). Individual leaf area, dry weight of total leaves, shoot and roots was determined by drying the tissue at 80 °C for 3 h, followed by incubation at 60 °C until constant weight was reached.

### Chlorophyll quantification

Leaf tissue (100 mg) was collected from fully expanded leaves (third leaf from the top) in four individuals from the different genotypes and treatments. Chlorophyll *a* and *b* were measured by a HPLC method^45^.

### Gas exchange measurements

Photosynthetic measurements were conducted in fully expanded leaves (third leaf from the top) using LI-COR 6400–40 (Li-6400, Li-Cor Inc., Nebraska, USA). Leaves were first equilibrated at a photon density flux of 1,500 µmol m^2^ s^−1^ (slightly higher than light saturation point) for at least 10 min and 370 µmol mol^−1^ of external CO_2_. Leaf temperature was maintained at 28 °C, and the leaf-to-air vapor pressure deficit was kept between 1 and 1.3 kPa. These conditions were kept constant for the determination of CO_2_ Net photosynthesis (*Pn*), stomatal conductance (*g*_*s*_), and water use efficiency (_i_WUE). WUE was calculated as the ratio between net photosynthesis and *g*_*s*_. CO_2_ response curves (A/Ci) were determined from 4 different plants per genotype and treatment. [CO_2_] in the leaf cuvette was set at 8 levels (100; 200; 400; 600; 800; 1000; 1200 and 1400 µmol m^−2^ s^−1^).

The relation between A and Ci was fitted with the software Photosyn Assistant (Dundee Scientific). The light saturated rates of electron transport (J_max_), maximal rate of carboxylation (V_Cmax_), and Triose Phosphate Utilization (TPU), were calculated using the Photosyn Assistant software (Dundee Scientific)^46^. CO_2_ compensation point (CCP), used as an estimative of photorespiration^35^, is the [CO_2_] at which oxygenation proceeds at twice the rate of carboxylation causing photosynthetic uptake of CO_2_ to be exactly compensated by photorespiratory CO_2_ release. It was estimated from the slope of the CO_2_ response curves at the lowest CO_2_ concentration^47^.

### Protein, ammonium, proline and betacyanins analysis in leaves

Total protein, ammonium (${{\rm{NH}}}_{4}^{+}$) and proline contents were determined under HN and LN conditions in leaves of the three Quinoa genotypes studied. Bradford assay^48^ was used for protein quantification on leaves using bovine serum albumin as a standard. ${{\rm{NH}}}_{4}^{+}$ was determined according to Forster^49^. Absorbance was measured at 660 nm in a spectrophotometer (Infinite 200 Pro, Tecan, Männedorf, Switzerland). Proline was determined using the method developed by Bates *et al*.^50^. The absorbance was measured at 520 nm. Betacyanins were extracted in water and pigment content in the solutions was determined by spectrophotometric determination at 536 nm. The betacyanin content of the plant aqueous extracts was estimated according to Abderrahim *et al*.^51^.

### Nitrate reductase and Glutamine synthetase activity

Both, Nitrate reductase (NR) and Glutamine synthetase (GS) catalyze the limiting steps in the reduction of NO^3−^ to ${{\rm{NH}}}_{4}^{+}$ (primary assimilation), and the ${{\rm{NH}}}_{4}^{+}$ incorporation into amino acids, respectively. NR activity (EC 1.6.6.1) was measured in mature leaves according to Kaiser and Lewis^52^. GS activity (EC 6.3.1.2) was measured by the formation of γ-glutamyl hydroxamate using the transferase assay^53^.

### Quantitative PCR

RNA was extracted from young and mature leaves using RNeasy_Mini kit (Qiagen), with three biological replicates. First strand cDNA was synthesized from 1 μg of total RNA with PrimeScript™ RT reagent Kit (Takara)^54^.

The mRNA sequences of the Quinoa genes were obtained from the Phytozome Database (https://phytozome.jgi.doe.gov/pz/portal.html). Gene^−^specific primers for *NR*, *GS*, *ASS1* and *AMT1*,*1* were designed using Premier 5.0 software (http://www.premierbiosoft.com/primerdesign) to have melting temperatures of 60 °C and generate PCR products of approximately 100–200 bp. The tubuline elongation factor (*CqHK1*) was used as endogenous control in order to normalize experimental results. Primers and locus name were:

*CqHK1*: Forward 5′-GTACGCATGGGTGCTTGACAAACTC-3′, Reverse 5′-TCAGCCTGGGAGGTACCAGTAAT-3′ (AUR62020772); *CqNR*: Forward 5′-AGGACTGGACCATTGAGGTG-3′, Reverse 5′-GCTGCAGAACCCCAATTAAA-3′ (AUR62004699), *CqGS2* Forward 5′-TCCATGTTTGATGCTGGCCT-3′, Reverse 5′-TGCAAATAGGGGTGCCTCTG-3′ (AUR62017693), *CqASS1* Forward 5′-AGGCTTTGACCCTTGATCGG-3′, Reverse 5′-CCATGGACTCACGAAGAGGG-3′ (AUR62017693), *CqAMT1*,*1* Forward 5′-CACTAGGGGAGCCGAAAGCTA-3′ Reverse 5′-TCCGTCCGTGCTAAGAACAC-3′ (AUR62035890).

PCR reaction contained 10 uL 2X SYBR Green QPCR master mix (Agilent Technologies), 50 ng cDNA, and 0.45 µM (final concentration) of each primer, in a final volume of 20 µl. Real-time PCR reactions were run at the Agilent Mx3000P QPCR System (Agilent Technologies). The PCR conditions were as follow: initial denaturing of 3 min at 95 °C, followed by 40 PCR cycles of 30 seconds at 95 °C, 18 seconds at 60 °C and 2 seconds at 60 °C, and a final extension cycle of 15 seconds at 95 °C, 1 second at 25 °C, 15 seconds at 60 °C, and 1 second at 95 °C. The comparative 2^−ΔΔCT^ method was used to quantify the relative abundance of transcripts Livak and Schmittgen 2001^55^.

### Seeds characteristics

Seed number per m^−2^ and weight of 1000 seeds were obtained from the yield for each genotype and treatment. Seed weight was determined by measuring the weight of 1000 oven-dried seeds and seed number per m^2^ by counting the number of seeds per square meter. N content in grains was determined by grinding and oven-drying overnight at 80 °C. One hundred milligram was used to quantify N^56,57^.

### Measurements of free amino acid levels in seeds

Free amino acids were extracted from seeds as previously described by Hacham *et al*.^58^. Approximately 200 mg of tissue was homogenized by mortar and pestle in the presence of 600 μl of water:chloroform:methanol (3:5:12, v/v). After a short centrifugation (10000 rpm), the supernatant was collected and the residue was extracted with 600 μl of the same mixture. The two supernatants were combined. Chloroform (300 μl) and water (450 μl) were added, and the resulting mixture was centrifuged again. The upper water-methanol phase was collected, dried, and dissolved in 200 μl of water. The concentration of free amino acids was determined using *O*-phthalaldehyde reagent, followed by measuring the 335/447 nm fluorescence. The composition of amino acids was determined by loading a 66-nmol sample of total free amino acids on an Amino Quant Liquid Chromatograph (Hewlett-Packard, Palo Alto, CA).

### Statistical analysis

Statistical analyses were performed using a two-way ANOVA, with genotypes and N supply as factors, followed by a Tukey *post hoc* analysis at a *P* < 0.05. Linear Pearson’s correlation coefficient (r) was used to examine the correlations between yield and the physiological parameters evaluated. All the statistical analyses were performed using the STATISTICA 6.0 software.
